# Pattern of systemic lupus erythematosus in Egyptian patients: the impact of disease activity on the quality of life

**DOI:** 10.4314/pamj.v6i1.69084

**Published:** 2010-08-23

**Authors:** Sliem Hamdy, Tawfik Gamal, Khalil Khalil A, Ibrahim Nagwa

**Affiliations:** 1 Department of Internal Medicine, Faculty of Medicine, Suez Canal University, Egypt

**Keywords:** Systemic lupus erythematosus, SLE, quality of life, SLE Disease Activity Index, Short Form–36 health questionnaire

## Abstract

**Introduction:**

Systemic lupus erythematosus (SLE) afflicts young people disproportionately, often at a crucial time in their lives when they are trying to establish relationships, start families and launch careers. As a result, persons with SLE may experience a wide range of physical and psychosocial problems that are not always fully captured by descriptions of the disease’s physiological consequences alone.

**Methods:**

In order to characterize the spectrum of the effects of SLE with regards to disease activity and its impact on the quality of life (QoL), a case control study involving 59 SLE Egyptian patients (mean age 28.6 years, 94.9% females) and 20 healthy controls was undertaken. Disease activity was measured by SLE Disease Activity Index (SLEDAI), and quality of life was measured by Short Form–36 health questionnaire (SF-36).

**Results:**

Mucocutaneous and hematological manifestations were present in most of the patients and arthralgia in half of them. All domains of SF-36 including general health, physical functions, physical limitations, energy/fatigue, emotional well-being, pain, social functions, and health changes were significantly lower in SLE patients compared to controls. Except for emotional limitations, all domains were correlated with disease activity and low in class IV-V lupus nephritis.

**Conclusion:**

Physicians should focus on QoL and how to improve it; health education regarding the negative impact of disease activity on the patients should be given attention. The results of QoL studies help physicians to understand and provide better support to SLE patients beside rapid meticulous control of disease activity.

## Introduction

Systemic lupus erythematosus (SLE) is a chronic autoimmune disease affecting almost all organ systems. It is characterized by exacerbations (or flares) of disease activity and disease damages. Measures of disease activity include; SLE disease activity index (SLEDAI) [1], British Isles Lupus Assessment Group (BILAG) disease activity index [2] and SLE Activity Measure (SLAM) [3].

However, in addition to disease activity and damages, other important consequences of the disease include changes in Quality of Life (QoL) affecting employment and social functioning. Therefore, in an effort to improve assessment of outcomes in SLE, the outcome measures in rheumatology clinical trials group has recommended that trials of SLE include outcome measures of QoL, adverse events and economic costs, in addition to measures of disease activity and damages [4].

The term health related QoL (HRQoL) refers to those aspects of life which are affected by health e.g. functional status, and excludes other determinants of QoL e.g.: income, job security or living conditions [5]. Measuring of HRQoL provides patients with an opportunity to participate more fully in their treatment and ultimately facilitate better communication with the multi-disciplinary team of health professionals involved in their care [6]. In addition to more objective clinical indicators of disease, measurement of HRQoL, allows for a more comprehensive assessment and in some cases may prove to be a more sensitive indicator of treatment response than measures of disease activity or damages [4,5].

The most commonly used measure of HRQoL is the short form (SF)-36. The SF-36 is a generic, 36-item self-report questionnaire. It was designed to be used in a variety of conditions, populations, and settings. The SF-36 has been shown to be a valid and reliable instrument in SLE and has been used in numerous studies in SLE [7].

In order to evaluate QoL in rural and urban areas in Lebanon, the SF-36 health survey was adapted into Arabic [8]. In Egypt, reliability of an Arabic version of the SF 36-Item health survey and its equivalence to the US-English version was performed by Abdul-Mohsin et al. [9].

On the basis of the hypothesis (SLE is a disease characterized by a variety of clinical manifestation with changes of its activity over time, and the quality of life of SLE patients during disease activity is low), the present study was designed with four objectives: 1) Studying the clinical and biochemical pattern of SLE in Egyptian patients; 2) Assess different aspects of quality of life in terms of physical, mental, psychological and social aspects; 3) Assess disease activity using SLEDI score; 4) Assess factors affecting quality of life including disease activity and renal involvement that were confirmed by previous records of kidney pathology.

## Methods

The present study was conducted as a descriptive case control study on 59 of SLE patients who registered at SLE special outpatient clinic in the Suez Canal University Hospital from January 2008 to April 2009. All fulfilled the Criteria of Classification of the American College of Rheumatology (ACR) for diagnosis of SLE [10]

Twenty healthy adults matched for age and gender were considered as a control group. They were defined by self-report and confirmed by physician observations. The control group was added to be compared with SLE group regarding QoL assessment.

The sample size was calculated according to the following equation [11]: N=(Za) 2 X PQ/D2: Where: N= number of subjects. Za = the value of standard normal distribution for type I error probability for the sided test and equals 1.96; P=prevalence of poor QoL of SLE patients=81%. Q=1-P, D2=the accuracy of estimate= 0.01. According to the calculations, the sample size was estimated at 59 patients.

At the time of HRQoL assessment, all patients were subjected to the following: 1) Complete medical history and clinical examination, 2) Outcome measures and assessment questionnaires: Disease activity was measured by SLEDAI score and measure of QoL was done by Medical Outcomes Study SF-36, which is a generic measure that is applicable in a variety of conditions, including SLE.

**Measuring disease activity using SLEDAI**

SLEDAI has 24 descriptors representing 9 organ systems affected by SLE. For every organ, score points are calculated. Points are based on a “weighted” index for lupus disease activity with 8 points for central nervous system and vascular system; 4 points for renal and musculoskeletal systems; 2 points for serosal, dermal and immunologic systems; 1 point for constitutional and hematological. The points were assigned if the descriptor is present at the time of the patient visit or within the preceding 10 days [1,12]. SLEDAI score = Sum of score points of 24 descriptors with minimum score = 0 and maximum score = 105. SLEDAI score grading: no, mild, moderate, and severe activities.

**Measuring the quality of life using SF-36 Health Survey questionnaire**

The Arabic [9] and the English [13] versions were used. The questionnaire included eight subscales (physical and social functioning, role limitations due to physical and emotional problems, mental health, energy/vitality, pain and general health perception) that can be summarized into two component scores; physical and mental component summary score.

**Laboratory investigations**

Laboratory investigation included complete blood picture and urine analysis. Kidney function tests, anti-nuclear antibodies (ANA), and anti-ds DNA antibodies (ELISA) were also performed.

**Pathological reports of kidney Biopsy**

Previous kidney biopsies were reviewed for all patients to estimate renal involvement and to determine the extent and severity of renal disease. Reports of biopsies were taken to be correlated with QoL of the patients. According to International Society of Nephrology – Renal Pathology Society Classification of Lupus Nephritis (2003), patients were classified into 6 classes: class I (minimal), class II (mesangial), class III (focal), class IV (diffuse), class V (membranous), and class VI (advanced sclerosis) [14,15].

**Ethical consideration**

Informed consent was obtained from all the adults. The aim and the value of the work were explained in a simplified manner for them. There was no harm inflicted on them. On the contrary, all had benefited from follow-up and the final results of the study. The study was approved by the ethical committee of faculty of medicine, Suez Canal University.

**Statistical Analysis**

The data were analyzed using parametric tests and presented in terms of mean, standard deviation (SD) of the mean and percentages. Statistical analysis was done using SPSS Ver. 11. Student-t, correlation coefficient, and Chi-square tests were used to evaluate the results. P value was set at <0.05 for statistically significant results and <0.0001 for highly significant results.

## Results

Out of 59 SLE patients, 56 were females (94.9%) and 3 patients (5.1%) were males. The age ranged from 16 to 42 years with a mean of 28.6±6.6 years. The mean disease duration was 5.6±3.4 years. There were no differences between cases and controls for demographic characteristic items (Table 1).

For clinical and biochemical findings (Table 2), malar rash was presented in 91.5% of the patients, photosensitivity was presented in 83.1% and discoid rash was presented in 20.3%. Eighty five percent (85%) of the patients were presented with mucosal ulcers. Arthritis was present in 52.5% of the SLE patients, while serositis (cardiopulmonary involvements) was present in 44.1% of them. 10.2% of the patients had had persistent proteinuria >0.5 grams/day or greater than 3+, 23.7% had cellular casts, and 54.2% had both proteinuria and urinary casts. About 51% of the patients had anemia, 55.9% had leucopenia and 33.9% had thrombocytopenia. 73% had elevated DNA and 6.8% had positive both anti-DNA and anti-phospholipids Abs.

According to SLEDAI score 51% of the SLE population in our study had mild activity and 27.2% had moderate to severe activity. No activity was present in 13%, while 16% had from moderate to severe activity.

Regarding renal pathology classification of lupus nephritis, 12% of the patients had class I, 5.1% had class II, 57.6% had class III, 20.3% class IV and 5.1% had class V.

Table 3 shows the main domains of QoL measured by SF-36 in SLE patients compared to control. All domains of SF-36 including general health, physical functions, physical limitations, energy/fatigue, emotional well-being, pain, social functions, and health changes were lower in the SLE patients compared with control except emotional limitations.

Table 4 shows that all domains of SF-36 including general health, physical functions, physical limitations, emotional limitations, energy/fatigue, emotional well-being, pain, social functions, and health changes were negatively correlated with grades of disease activity as detected by SLEDAI.

Table 5 shows the mean score of each domains of QoL measured by SF-36 according to renal pathology classification of lupus nephritis in SLE patients. All domains of SF-36 including general health, emotional limitations, physical limitations, energy/fatigue, emotional well-being, pain, social functions, and health changes were lower in class IV-V the SLE patients compared with other classes except physical functions.

Table 6 shows the correlation between QoL score measured by SF-36 and activity score measured by SLEDAI in SLE patients. The correlation was assessed through Pearson correlation. There are significant correlations between general health, pain and social functions domains in QoL score and activity score measured by SLEDAI in SLE patients.

## Discussion

In the evaluation of patients with SLE it is important to measure not only disease activity (which is potentially reversible with treatment) and damage (which is permanent and can be due to the disease or treatment) but also the patients’ perspective. This is because the disease is likely to have a significant impact on the physical, social and psychological aspects of the patient health and QoL [16].

The present clinical and biochemical data had revealed that, mucocutaneous and hematological manifestations were present in most of the patients, while arthralgia was present in half of them. Many variations were seen in other studies. Ch et al studied 1082 SLE patients; 70% of the patients were characterized predominantly by mucocutaneous manifestations [17]. Sultan et al reported, out of 305 SLE patients, 2.5% only had hematological disorders [18]. In the cohort study of Alarcon et al., the first ACR criteria fulfilled were arthritis (34.5%) and photosensitivity (18.2%) [19]. In the Sultanate of Oman, Abdwani et al followed 50 children with lupus; the initial manifestations were arthritis or arthralgia (76%), cutaneous (70%), hematological (68%), and renal (64%) [20]. Additionally, Nazarinia et al found that out of 410 patients in Iran, 78% had hematological disorders and 38% had serositis [21]. Wallace and Tumlin, documented that 78% of SLE patients had anemia, and 30% had thrombocytopenia [22]. These variations may be due to different sample sizes, different patients’ ages, variable diseases durations, unreported recent or mild cases, and seasonal, regional or racial variations.

Traditionally, SLE is a disease with great impact on all aspects of health status. QoL is increasingly being recognized as an important aspect of chronic diseases. Its measurement has traditionally relied on the use of generic or disease-specific questionnaires. Generic questionnaires were developed for general use and may be used in a variety of diseases and populations. They allow for comparison with other groups and conditions and allow measurement of dysfunction for individuals experiencing more than one condition. Therefore, general health instruments have been shown to be valid for measuring QoL in SLE patients. In contrast, disease-specific questionnaires are designed to measure outcomes in a specific disease [23]. Only recently have disease-specific instruments been developed for use in SLE and these are not yet widely used [16].

In our study, we observed a progressive decrease in all SF-36 scores. These progressive changes in HRQoL could be due to several factors, such as SLE progression along the years, continuously coping with a chronic illness, and practical management items that may be required (frequent medical visits, laboratory examinations, etc.). Other possible explanations are that, most of our SLE patients were young adults females, and in ages at which physical, psychological and social stability had not yet been reached. The disease started at a crucial time in their lives when they were trying to establish relationships, start families and launch careers. As a result, patients experienced a wide range of physical, psychological and social problems.

In addition, all domains of SF-36 including general health, physical functions, physical limitations, emotional limitations, energy/fatigue, emotional well-being, pain, social functions, and health changes were correlated with disease activity as detected by SLEDAI.

Abu-Shakra M et al. found similar results. SF-36 scores were correlated with BILAG and SLEDAI scores, suggesting divergent construct validity of the SF-36 meaning it offered an independent assessment of the impact of SLE [24]. Also, Thumboo et al. reported that improvements in SF-36 scores correlate with decreases in disease activity and damage. Decrements in SF-36 scores reflect end stage renal disease and immunosuppressive use [25]. Various degrees of correlation between SLE disease activity and HR QoL were documented by some authors. In a cross-sectional analysis, Fortin PR et al. found that SLAM-R, correlated with most subscales of the SF-36. In a longitudinal analysis, both disease activity instruments (SLAM-R and SLEDAI) were associated with changes in the SF-36 [26]. Sutcliffe N et al. found that higher disease activity correlated with worse physical and emotional function, pain, and general health [7].

Khanna et al. used the World Health Organization Quality of Life-Bref (WHOQoL-Bref) to evaluate the relationship between disease activity and health status in the Indian subcontinent. The WHOQoL-Bref includes an environment domain, which the authors wanted to capture. They found that physical and psychological QoL are impaired with active lupus, whereas social and environmental QoL do not correlate with disease activity [27]. Simone et al reported that patients with SLE and active renal disease concurrently experience a slightly poorer QoL than those without renal disease, especially in the physical domains [28]. Lai et al found that low-grade inflammation as reflected by low serum albumin and hemoglobin concentrations were associated with impaired HRQOL in patients with SLE, independent of other socio-demographic and clinical variables [29]. Study by Strand et al showed that, the impact of SLE was evident - with decrements in HRQoL similar to those reported by patients with inflammatory arthritis, chronic congestive heart failure and post myocardial infarction despite differing disease activity and baseline disease [30].

To evaluate a cross-country comparison, Panopalis et al. (2005) evaluated the Short Form-36 General Health Survey (SF-36) scores done annually over 4 years in 231 patients from Canada, 269 from the United States, and 215 from the United Kingdom. They found that the physical and mental well-being components did not differ significantly between countries [31]. Whether the equivocal results on the relationship with disease activity and quality of life measures will be resolved with more uniform use of activity and health status indices remains to be seen. Larger studies including cohorts from diverse geographic regions are also needed for comparisons to better understand lupus variability.

In addition, we reported that the QoL of SLE patients as measured by SF-36 was poorer than healthy control in all domains except emotional limitations. Both physical and mental component summary scores of the SF-36 were reduced in our studied patients compared with controls. In SLE great variability in all the subscales was observed. Significant correlations between physical and mental components and different subscales were observed in patients but not in controls. In SLE, HRQoL tended to worsen with age. Higher disease activity and damage were associated with significantly lower HRQoL and worsening of SLE leads to a further decline. The same observations were detected by Rinaldi et al. They examined HRQoL in Italian patients with SLE and compared it with that of healthy people and investigated relationships among different dimensions and subscales of a generic health status measure [32]. They used SF-36 as we do and it was applied in a cohort of 126 consecutive SLE patients and 96 healthy controls. Current results were also in agreement with other studies in different patients age groups [33,34].

## Conclusion

Most of SLE studied patients were characterized predominantly by mucocutaneous and hematological manifestations. The QoL of SLE patients with renal involvement as measured by SF-36 was poorer than healthy control in all domains except emotional limitations. There are significant negative correlations between general health, pain and social functions domains in QoL score and activity score measured by SLEDAI in SLE patients. Physicians should focus on QoL and how to improve it in relation to bio-psychosocial approach, (health education regarding the negative impact of disease activity on the patients is required). The results of quality of life studies help physicians understand and give better support to SLE patients beside rapid meticulous control of disease activity. Further researches on the factors affecting QoL in SLE patients, including drugs are required.

## Competing interests

None declared.

## Authors’ contributions

HS: study concept, statistical analysis, manuscript writing and review. GT: study design, manuscript writing and review. KK: study concept, editing and review. NI: data collection and statistical analysis.

## Figures and Tables

**Table 1:** The socio-demographic characteristics of case and control groups

**Item**	**Case Group**	**(N=59)**	**Control Group**	**(N=20)**	**X^2^**	**P**
	**N**	**%**	**N**	**%**		
**Age**						
16-29 years	25	42.80%	9	45%	5.73	n.s
30-42	34	57.60%	11	55%		
**Gender**						
Male	3	5.10%	1	5%	6.8	n.s
Female	56	94.90%	19	95%		
**Marital status**						
Single	11	18.60%	3	15%		
Married	41	69.50%	15	75%	4.7	n.s
Widow	4	6.70%	1	5%		
Divorced	3	5.20%	1	5%		
**Educational status**						
Illiterate	2	3.40%	1	5%		
Write & read	5	8.50%	2	10%	5.3	n.s
Moderate	37	62.70%	11	55%		
High	15	25.40%	6	30%		
**Occupational status**						
Officers	21	35.60%	6	30%		
Workers	12	20.40%	5	25%		
Housewives	12	20.40%	6	30%	3.75	n.s
Students	4	6.70%	1	5%		
Others	10	16.90%	2	10%		

n.s: non-significant

**Table 2:** Clinical and laboratory findings in studied SLE population

**Items**	**No**	**%**
**Mucocutanous manifestations**		
Malar rash	54	91.5
Discoid rash	12	20.3
Photosensitivity	49	83.1
Oral ulcers	50	84.7
**Arthritis**	31	52.5
**Serositis (pleurisy, pericardium rub)**	26	44.1
**Renal findings**		
Non	7	11..9
Persistent proteinuria >0.5 grams/day or greater than 3+	6	10.2
Cellular casts	14	23.7
Both	32	54.2
		
**Neuropsychiatric manifestations**		
Non	41	69.5
Seizures	10	16.9
		
Psychosis	1	1.7
Persistence unexplained headache	7	11.9
**Hematological manifestations**		
No	3	5.1
Anemia	30	50.8
Leucopenia (<4000/mm)	33	55.9
Lymphopenia (<1500/mm)	3	5.1
Thrombocytopenia (<100.000/mm)	20	33.9
Anti-DNA & anti-phospholipids Abs negative	12	20.3
Anti-DNA Abs positive	42	73.9
Anti-DNA & anti-phospholipids Abs positive	4	6.8
ANA positive	59	100

**Table 3:** Main domains of QoL measured by SF-36 in SLE patients and controls

	**SLE**			**Control**					
	**Mean**	**SD**	**Mean**	**SD)**	**p-value**
General health	44.8	11.8	64.2	16.6	<0.001
Physical functions	55.5	20.1	71	25.1	<0.001
Physical limitations	26.3	37.3	75	24.4	<0.001
Emotion limitations	71.1	28.2	73.3	17.5	n.s
Energy/fatigue	42.5	15.9	68	23.8	<0.001
Emotion well-Being	36.9	15.4	59.2	24.1	<0.001
Pain	39.2	28.2	64.5	13.2	<0.001
Social functions	55.5	23.2	71	27.8	<0.001
Health change	64.8	24.2	79	16.5	<0.001

QoL: Quality of Life, SLE: Systemic Lupus erythematosus, SF: Short Form–36 health questionnaire

**Table 4:** The socio-demographic characteristics of case and control groups

**Item**	**Case Group**	**(N=59)**	**Control Group**	**(N=20)**	**X^2^**	**P**
	**N**	**%**	**N**	**%**		
**Age**						
16-29 years	25	42.80%	9	45%	5.73	n.s
30-42	34	57.60%	11	55%		
**Gender**						
Male	3	5.10%	1	5%	6.8	n.s
Female	56	94.90%	19	95%		
**Marital status**						
Single	11	18.60%	3	15%		
Married	41	69.50%	15	75%	4.7	n.s
Widow	4	6.70%	1	5%		
Divorced	3	5.20%	1	5%		
**Educational status**						
Illiterate	2	3.40%	1	5%		
Write & read	5	8.50%	2	10%	5.3	n.s
Moderate	37	62.70%	11	55%		
High	15	25.40%	6	30%		
**Occupational status**						
Officers	21	35.60%	6	30%		
Workers	12	20.40%	5	25%		
Housewives	12	20.40%	6	30%	3.75	n.s
Students	4	6.70%	1	5%		
Others	10	16.90%	2	10%		

n.s: non-significant

**Table 5:** Mean score of each domains of QoL measured by SF-36 according to renal pathology classification of lupus nephritis in SLE patients

**Domains**	**Class I-II**	**Class III**	**Class IV-V**	**p-value**
General health	46.9±13.2	43.3±10.5	40.0±6.7	<0.001
Physical functions	57.5±22.4	57.0±15.1	54.3±21.7	n.s
Physical limitations	35.3±4.4	17.5±3.4	11.7±6.5	<0.001
Emotion limitations	96.7±10.5	87.3±28.4	84.4±25.3	<0.001
Energy/fatigue	46.2±6.7	38.3±4.0	36.0±3.5	<0.001
Emotion well-Being	39.6±8.5	33.6±9.3	32.8±9.0	<0.001
Pain	46.6±3.7	31.5±2.5	25.8±3.9	<0.001
Social functions	59.0±22.1	51.9±27.3	50.2±20.5	<0.001
Health change	69.1±26.9	60.0±20.7	57.5±16.9	<0.001

QoL: Quality of Life, SLE: Systemic Lupus Erythematosus SF-36: SF: Short Form–36 health questionnaire

**Table 6:** Correlations between QoL score measured by SF-36 and activity score measured by SLEDAI in SLE patients

**Domains**	**Domains Pearson Correlation Coefficient**	**p-value (2-tailed)**
General health	-0.279	0.033*
Physical functions	0.164	0.214
Physical limitations	-0.224	0.088
Emotion limitations	-0.07	0.598
Energy/fatigue	-0.165	0.211
Emotion well-Being	-0.035	0.79
Pain	-0.32	0.014*
Social functions	-0.3	0.021*
Health change	-0.23	0.08

QoL: Quality of Life, SLEDAI: SLE disease activity index, SLE: Systemic Lupus Erythematosus SF-36: SF: Short Form–36 health questionnaire
